# The complete mitochondrial genome of *basiprionota bisignata* (Boheman, 1862) (Coleoptera: Chrysomelidae)

**DOI:** 10.1080/23802359.2022.2047117

**Published:** 2022-03-02

**Authors:** Shaochuan Cheng, Huimin Yuan, Ting Wang, Kai Hu

**Affiliations:** a Guizhou Academy of Forestry, Guiyang, P. R. China; b Forestry Administration of Yinjiang County, Tongren, P. R. China

**Keywords:** Mitogenome, phylogenetic analysis, Cassidinae, *Basiprionota*

## Abstract

The complete mitochondrial genome of *Basiprionota bisignata* (Boheman, 1862) (a species of leaf beetles) was successfully sequenced, annotated, and analyzed in this study. This mitochondrial genome is a circular DNA molecule of 16,069 bp in size with 78.5% AT content, including 13 protein-coding genes (PCGs), 2 ribosomal RNA genes (rRNAs), 22 transfer RNA genes (tRNAs), and an AT-rich region (control region). The gene order is consistent with the putative ancestral arrangement of insects. All PCGs are initiated by ATN (A/T/C/G) condons and terminated with TAA/G or their incomplete form single T-. All tRNAs can be folded into common clover leaf secondary structures, except for *trnS1*. The phylogenetic tree was reconstructed using maximum likelihood analysis, and the topology recovered the monophyly of Cassidinae and the sister relationship between *Basiprionota* and the clade (*Thlaspida* + *Aspidomorph*).

The genus (*Basiprionota* Chevrolat, 1837) belongs to the subfamily Cassidinae of Chrysomelidae, comprising 63 species distributed in Oriental region and border parts of Palearctic and Australopapuan regions (Borowiec 1999; Borowiec and Świętojańska 2002). In this study, we sequenced the complete mitochondrial genome of *Basiprionota bisignata* (Boheman, 1862), the first representative of *Basiprionota,* and performed a phylogenetic analysis among Chrysomelidae with the available mitogenomic sequences.

The samples of *B*. *bisignata* (specimen number: GZAF-2021-CC1000) were obtained from Yinjiang County (E108.3666, N28.0846), Guizhou province, China by Huimin Yuan and Ting Wang in May 2021, and stored in Insect Museum of Guizhou Academy of Forestry (URL, Kai Hu and 18792617323@163.com), Guiyang. Total genomic DNA was extracted from an adult’s thoracic muscle using the DNeasy Blood and Tissue Kits (Qiagen, Valencia, CA). Based on the high-throughput Illumina Hiseq X platform, total genomic DNA was sequenced. The raw data (3.16 Gb) were assembled using NOVOPlasty version 4.3.1 (Dierckxsens et al. 2017) with *cox1* sequence from *Aspidomorpha difformis* (GenBank accession no. MK049862) as the initial seed. The complete mitochondrial genome of *B. bisignata* was annotated by MITOZ version 1.04 (Meng et al. 2019). All 13 protein-coding gene sequences were aligned using MAFFT version 7.394 (Kuraku et al. 2013) with L-INSI-I strategy. Maximum likelihood (ML) analysis was conducted using IQ-TREE version 1.6.3 (Nguyen et al. 2015) with the optimal model (GTR + I + G for Subset1 (*nad3* and *atp6*), Subset3 (*cox1*, *cytb*, *cox3*, and *cox2*), and Subset4 (*nad1*, *nad4L*, *nad4*, and *nad5*); TRN + I + G for Subset2 (*nad6* and *atp8*); TVM + I + G for Subset5 (*nad2*)) were determined by PartitonFinder2 (Lanfear et al. 2017).

The complete mitochondrial genome of *B. bisignata* is 17,116 bp in length, containing 13 protein-coding genes (PCGs), 2 ribosomal RNA genes (rRNAs), 22 transfer RNA genes (tRNAs), and an AT-rich region (control region). The gene order of the newly sequenced mitochondrial genome is consistent with the putative ancestral arrangement of insects (Clary and Wolstenholme 1985; Cameron 2014). The AT content of the mitochondrial genome is 78.5% (A = 42.8%, T = 35.7%, C = 12.8%, and G = 8.7%), which has a strong AT nucleotide bias. Most PCGs (*nad2*, *cox1*, *atp8*, *atp6*, *nad3*, *nad4*, *nad4L*, *nad6*, *cytb*, and *nad1*) share typical stop termination TAA/G, whereas *cox2*, *cox3*, and *nad5* end with incomplete form single T–. Furthermore, all PCGs use ATN (A/T/C/G) as start codon. Length of the 22 tRNAs ranges from 60 bp (*trnE*) to 68 (*trnM*). All tRNAs can be folded into common clover-leaf secondary structures, except for *trnS1*, in which the dihydrourine (DHU) arm formed a simple loop. The size of *16SrRNA* and *12SrRNA* is 1,259 bp and 740 bp, respectively. The AT-rich region is located between *12SrRNA* and *trnI*, which is 2,515 bp in length with an AT content of 82.8%.

Here, based on the nucleotide data of 13 PCGs from 27 Chrysomelidae species and two outgroup taxa from Cerambycidae, we reconstructed the ML phylogenetic tree (Figure 1). The phylogenetic relationships among Chrysomelidae are ((Cassidinae + (Eumolpinae + (Chlamisinae + Clytrinae))) + ((Galerucinae + Chrysomelinae) + (Bruchinae + (Donaciinae + Criocerinae)))). In the phylogenetic tree, each subfamily forms a monophyletic cluster with strongly support (BS ≥ 98), consistent with some previous studies (Gómez-Zurita et al. 2008; Nie et al. 2020). In Cassidinae, the relationships among included genera are inferred as ((*Octodonta* + *Brontispa*) + (*Basiprionota* + (*Thlaspida* + *Aspidomorpha*))).

**Figure 1.**
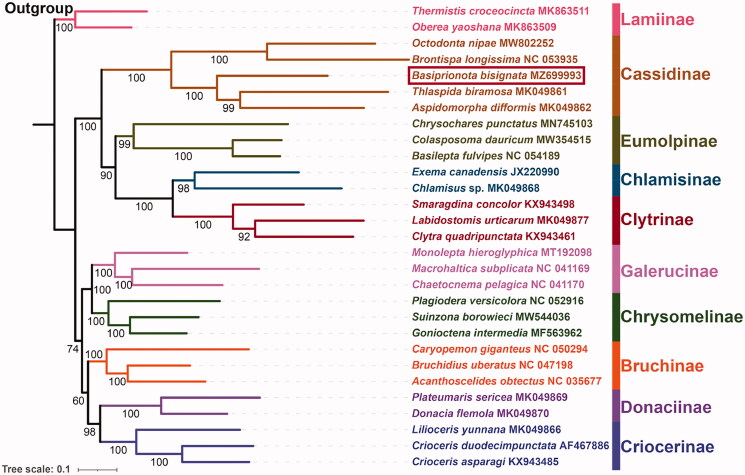
Maximum likelihood phylogenetic tree for Chrysomelidae based on the nucleotide sequence data of 13 PCGs from *B*. *bisignata* and other 26 species belonging to nine related subfamilies of Chrysomelidae. The number on each node indicates bootstrap support value (BS).

## Data Availability

The genome sequence data that support the findings of this study are openly available in GenBank of NCBI at (https://www.ncbi.nlm.nih.gov/) under the accession no. MZ699993. The associated BioProject, SRAs, and Bio-Sample numbers are PRJNA759086, SRR15686511-SRR15686512, and SAMN21155885, respectively.
